# Effects of palmitic acid on localization of embryo cell fate and blastocyst formation gene products

**DOI:** 10.1530/REP-21-0354

**Published:** 2022-01-13

**Authors:** Michele D Calder, Robert Chen, Anastasia MacDonald, Zoe MacNeily, Zuleika Chin Lai Leung, Samira Adus, Shiyu Cui, Dean H Betts, Basim Abu Rafea, Andrew J Watson

**Affiliations:** 1 Department of Obstetrics and Gynaecology, The University of Western Ontario, London, Ontario, Canada; 2 Department of Physiology and Pharmacology, The University of Western Ontario, London, Ontario, Canada; 3 The Children’s Health Research Institute – Lawson Health Research Institute, London, Ontario, Canada

## Abstract

As obese and overweight patients commonly display hyperlipidemia and are increasingly accessing fertility clinics for their conception needs, our studies are directed at understanding the effects of hyperlipidemia on early pregnancy. We have focused on investigating palmitic acid (PA) and oleic acid (OA) treatment alone and in combination from the mouse two-cell stage embryos as a model for understanding their effects on the mammalian preimplantation embryo. We recently reported that PA exerts a negative effect on mouse two-cell progression to the blastocyst stage, whereas OA co-treatment reverses that negative effect. In the present study, we hypothesized that PA treatment of mouse embryos would disrupt proper localization of cell fate determining and blastocyst formation gene products and that co-treatment with OA would reverse these effects. Our results demonstrate that PA treatment significantly (*P*  < 0.05) reduces blastocyst development and cell number but did not prevent nuclear localization of YAP in outer cells. PA treatment significantly reduced the number of OCT4^+^ and CDX2^+^ nuclei. PA-treated embryos had lower expression of blastocyst formation proteins (E-cadherin, ZO-1 and Na/K-ATPase alpha1 subunit). Importantly, co-treatment of embryos with OA reversed PA-induced effects on blastocyst development and increased inner cell mass (ICM) and trophectoderm (TE) cell numbers and expression of blastocyst formation proteins. Our findings demonstrate that PA treatment does not impede cell fate gene localization but does disrupt proper blastocyst formation gene localization during mouse preimplantation development. OA treatment is protective and reverses PA’s detrimental effects. The results advance our understanding of the impact of FFA exposure on mammalian preimplantation development.

## Introduction

The World Health Organization defines obesity as a BMI of 30 kg/m^2^ or greater (WHO 2000) and obesity is reaching epidemic proportions. The incidence of diabetes, hypertension, heart disease and high serum cholesterol demonstrates a graded increase as BMI rises (Paeratakul *et al.* 2002, Knight *et al.* 2010). Obese females face many challenges including menstrual irregularity, endometrial pathologies, higher miscarriage rates and infertility (Broughton & Moley 2017). As BMI increases, women experience increased time-to-pregnancy and overall decreased fecundity (Wise *et al.* 2010). Furthermore, when obese women conceive, their pregnancies have increased incidence of preterm birth, high- and low-birth weight infants, increased risk of congenital anomalies (Knight *et al.* 2010, Schummers *et al.* 2015, Stang & Huffman 2016), pre-eclampsia and gestational diabetes (Stang & Huffman 2016). Assisted reproductive technologies (ARTs) are increasingly being required to achieve successful pregnancy in sub-fertile obese couples. Increasing BMI is positively correlated with pregnancy loss following ART treatment (Fedorcsák *et al.* 2004).

Obesity is accompanied by increased serum (Gaudet *et al.* 2012) and follicular fluid palmitic acid (PA) levels (Niu *et al.* 2014, Valckx *et al.* 2014). PA is a 16-carbon fully saturated fatty acid that increases the synthesis of harmful lipids and impedes normal organelle functioning (Yuzefovych *et al.* 2010, Palomer *et al.* 2018). It is the most abundantly saturated fatty acid in the human body (Carta *et al.* 2017). PA is present in many foods including meat, dairy and palm oil (Carta *et al.* 2017). The dysregulation of PA levels has detrimental long-term effects such as atherosclerosis, neurodegenerative disorders and cancer (Fatima *et al.* 2019).

Oleic acid (OA) is an 18-carbon fatty acid with a single double bond (Lopez *et al.* 2014). It is the most abundant unsaturated fatty acid in human plasma (Palomer *et al.* 2018). OA can be obtained in the human diet through olive, canola and peanut oils (Lopez *et al.* 2014) and is a primary component of the Mediterranean diet which lessens disease incidence such as diabetes (Martınez-Gonzalez *et al.* 2008). OA decreases PA-induced apoptosis and ROS (Yuzefovych *et al.* 2010), decreases PA-induced inflammation and relieves ER stress (Palomer *et al.* 2018). We have recently reported that PA treatment reduces mouse blastocyst development in a concentration-dependent manner and alters ER stress pathway transcript levels, while co-treatment of OA with PA reversed these negative effects (Yousif *et al.* 2020). Interestingly, we observed a small percentage of PA (100 µM) treated two-cell stage embryos that still cavitate, but for this study, we were especially interested in those that were unable to cavitate and become blastocysts.

Mammalian preimplantation development is characterized by a series of cleavage cell divisions from fertilized oocyte to multi-cellular blastocyst (Watson & Barcroft 2001). The initial cleavages occur without concomitant embryo growth (Hardy *et al.* 1993), and eventually the cells compact and form a morula (Watson & Barcroft 2001, Pfeffer 2018). At the mouse 32-cell stage, a cavity begins to form, leading to the blastocyst stage and the specification of two distinct cell lineages: the TE and ICM (Watson & Barcroft 2001, Niwa *et al.* 2005).

OCT4 (POU5F1)is a pluripotency cell factor that gradually becomes restricted to the mouse ICM, acting to regulate transcription of target genes and maintain ICM pluripotency (Nichols *et al.* 1998). CDX2 is expressed in all cells at the 8-cell stage and gradually becomes restricted to the future trophectoderm in the blastocyst (Niwa *et al.* 2005). CDX2-deficient embryos have a poorly formed TE with deficiencies in maintaining tight junctions (Strumpf *et al.* 2005). OCT4 is downregulated when CDX2 reaches a specific threshold in mouse outer cells and collectively this asymmetry in cell lineage expression drives the formation of the ICM and TE (Niwa *et al.* 2005). CDX2 expression is induced by the transcription factor TEAD4 (Nishioka *et al.* 2009) and also requires Hippo pathway member YAP (Ota & Sasaki 2008). Failure to properly generate the first cell lineages can prevent the initiation of pregnancy (Piliszek *et al.* 2016).

Blastocyst development depends on the timely expression of critical gene families including: E-cadherin–catenin cell adhesion genes, the tight junction genes and Na/K-ATPase subunits (reviewed in Watson & Barcroft 2001, Bell *et al.* 2008). Na/K-ATPase develops the ionic gradient which drives the movement of water through aquaporins across the epithelium to form the fluid-filled blastocyst cavity (Watson & Barcroft 2001). The cell-to-cell adhesion provided by E-cadherin–catenin and tight junctional proteins such as ZO-1 inhibits the leakage of blastocyst fluid and maintains a polarized Na^+^/K^+^-ATPase distribution (Watson & Barcroft 2001). These events are interconnected, as interrupting expression or activity of Na/K-ATPase in embryos affects localization of ZO-1 (Violette *et al.* 2006, Madan *et al.* 2007).

Because of the many fertility challenges obese women face and the importance of preimplantation development to successful pregnancy (Knight *et al.* 2010), we have pursued an understanding of how preimplantation development may be affected by PA and OA exposure. To date, research has not explored the effects of PA and OA on cell fate gene product and blastocyst formation gene product localization during preimplantation development. The primary objective was to determine how PA and OA treatment, alone and in combination, will affect YAP, OCT4, CDX2, Na/K ATPase α-1, E-cadherin and ZO-1 localization in mouse preimplantation embryos. We hypothesized that PA treatment of mouse preimplantation embryos would disrupt normal cell fate gene product and blastocyst formation gene product localization and that co-treatment with OA would reverse these effects. Our findings demonstrate that PA treatment does not impede cell fate gene product localization but reduces cell margin localization of blastocyst formation proteins. OA co-treatment reverses these effects of PA treatment. The results advance our understanding of the possible impact of obesity and FFA exposure on mammalian preimplantation development.

## Materials and methods

### Animal source and ethics approval

CD1 mice were acquired from Charles River Canada (Saint-Constant, Quebec, Canada). The mice had free access to food and water while being housed in 12 h light: 12 h darkness cycles. All mice were handled according to the Canadian Council on Animal Care and Western University’s Animal Care and Use Policies (protocol #: 2018-075 to Dr Andrew J. Watson).

### Mouse superovulation and mating

Four- to six-week-old female CD-1 mice received a 5–7.5 international units (IU) intraperitoneal (i.p.) injection with pregnant mare’s serum gonadotropin (Folligon, Merck Animal Health, Canada). This was followed by a 5–7.5 IU i.p. injection of human chorionic gonadotrophin (Chorulon, Merck) 46–48 h later. Following the second injection, each female was placed in a cage with a single male CD-1 mouse (3–8 months of age) for mating overnight. The following morning, female mice were checked for presence of a seminal plug. Forty-six hours post-injection of hCG, female mice were sacrificed by CO_2_ asphyxiation (standard operating procedure). Their oviducts were collected and flushed with M2 flushing medium (Sigma–Aldrich). Flushed two-cell stage embryos were washed 3× in 50 μL drops of potassium simplex optimization medium with amino acids (KSOMaa Evolve, Zenith Biotech, Canada) for OCT4/CDX2 and cell counting experiments. Due to discontinuation of this product, YAP, Na/K ATPase α-1, E-cadherin and ZO-1experiments were conducted with KSOM+AA (IVL04, Caisson Laboratories, Smithfield, UT). After washing, embryos were distributed equally among experimental treatment groups and cultured in 20 μL drops under mineral oil (LiteOil LGOL-500, Cooper Surgical, Trumbull, CT) at a density of 1 embryo per μL for 46 h under a 5% CO_2,_5% O_2_ and 90% N_2_culture atmosphere.

### Free-fatty acid (FFA) preparation and embryo culture

A 20% FFA-free BSA (A6003, Sigma) was prepared in PBS, filter sterilized and used to conjugate with PA (P0500, Sigma) or OA (75090, Sigma) as outlined in Alsabeeh *et al.* (2018). Stock PA and OA solutions were prepared by solubilizing each FFA in RNAse-free water and NaOH at 70°C to create a 20 mM solution that was stored at −20°C. This stock was heated to 70°C and conjugated in a 1:3 (v/v) ratio with 20% BSA initially, followed by a dilution to 500 µM FFA solution in KSOM that was filtered and stored at 4°C. This results in a 2:1 molar ratio of PA or OA to BSA. The BSA control had a similar amount of added NaOH. All treatment groups thus contained 0.6% added fatty acid-free BSA final concentration.

### PA and OA concentration response and treatment experiments

PA and OA treatment concentrations were derived from concentration responsive experiments conducted and reported in our recent study (Yousif *et al.* 2020); 100 µM PA had a negative effect on blastocyst development and 100 µM OA was able to overcome these deficits. PA is in the low physiological range, as 300–400 µM PA was measured in the regular chow-fed mouse oviduct (Yousif *et al.* 2020). Third trimester women with normal glucose tolerance had serum levels of 122 µM PA and 83 µM OA (Chen *et al.* 2010) which increased in obese women with gestational diabetes to 151 and 104 µM, respectively. For each experimental replicate, treatment groups consisted of 2- to 4-cell stage mouse embryos randomly allocated to the following treatments: (1) control KSOMaa medium (120 μL BSA control in a total volume of 300 μL balance KSOM); (2) KSOMaa medium +100 μM PA (60 μL + 60 μL BSA control in 300 μL); 3) KSOMaa medium + 100 µM OA (60 μL + 60 μL BSA control in 300 μL) and 4) KSOMaa + 100 µM PA (60 μL) and 100 µM OA (60 μL in 300 μL). Embryos were cultured under a 5% CO_2_, 5% O_2_ and 90% N_2_ atmosphere at 37°C for 46 h to assess progression to the blastocyst stage. Blastocysts were defined as embryos with a visible fluid-filled cavity of any size and the proportion was calculated based on number of two-cell cultured. Following this assessment, embryos were fixed for application of indirect immunofluorescence localization of OCT4, CDX2, YAP, Na/K-ATPase ∝ 1 subunit, ZO-1 or E-cadherin combined with 4,6-diamindino-2-phenylindole (DAPI) nuclear staining, actin cytoskeleton staining with rhodamine phalloidin and confocal microscopy.

### Immunofluorescence and confocal microscopy

Following culture, embryos in each treatment were fixed and permeabilized in 2% paraformaldehyde in PBS for 30 min. Embryos were stored in PIPES/HEPES/EGTA and MgCl_2_ (PHEM, Schliwa & van Blerkom 1981) buffer at 4°C before staining. The preimplantation embryos were then blocked in 5% normal donkey serum (Jackson ImmunoResearch) with 0.01% Triton X-100 in PBS for at least 1 h at room temperature. Embryos were washed once in PBS. Then primary antibodies were added to antibody dilution buffer (ADB) consisting of PBS containing 0.5% normal donkey serum and 0.005% Triton-X and incubated overnight at 4°C. Antibodies included (1) mouse anti-YAP (sc-101199, Santa Cruz Biotechnology) diluted (1:100), (2) mouse anti-OCT3/4 (C-10, sc-5279, 1:50 Santa Cruz), (3) rabbit anti-CDX2 (ab76541, 1:100; Abcam), (4) rabbit anti-E-cadherin (#3195, Cell Signaling) or (5) rat anti-ZO1 (MABT11, EMD Millipore), diluted in ADB. Rabbit-sodium potassium ATPase alpha1 subunit antibody (ab237969, 1:100, Abcam) staining was performed completely in PHEM buffer (Betts *et al.* 1998). Donkey anti-mouse Alexa Fluor 488- (715-545-151, Jackson ImmunoResearch), donkey anti-rabbit fluorescein (711-095-152, Jackson), donkey anti-rat fluorescein (712-095-153, Jackson) or donkey anti-rabbit Alexa Fluor 555-conjugated secondary antibodies (A31572, Thermo Fisher Scientific) were added and incubated at 4°C overnight. Embryos were counterstained with final 1:1000 dilution of 1 mg/mL stock solution DAPI (D9542, Sigma) to stain all nuclei blue and 1:20 final dilution of 5 µg/mL stock solution of rhodamine phalloidin (P1951, Sigma) to stain the actin cytoskeleton red for 1 h at 37°C. Processed embryos were mounted on glass bottom dishes in drops of KSOMaa and covered with mineral oil (Sigma) to prepare for confocal imaging. Confocal microscopy was performed using a Zeiss LSM800 laser scanning confocal microscope (Carl Zeiss Microscopy). Increments of 5 µm were used to produce a Z-stack for each embryo. Laser strength settings were consistent between groups within a replicate. Z-stacks were saved as CZI files on Zen imaging program (Zeiss). Using the cell counter function, OCT4, CDX2, YAP and DAPI total nuclei were quantified manually in FIJI (open source softeare ImageJ available at https://imagej.net/software/fiji/). Nuclei were counted as positive if they had fluorescence substantially above cytoplasmic levels, cells that were dividing had increased cytoplasmic staining for OCT4 and were not counted as positive.

### Quantitation of Na^+^/K^+^ ATPase alpha1 subunit, E-cadherin and ZO1 fluorescence intensity

Na^+^/K^+^ ATPase alpha1 subunit, E-cadherin and ZO-1 fluorescence intensity were quantified using Zen and Ilastik (www.ilastik.org). From the embryo group Z-stacks czi files taken at 10×, rectangular boxes were drawn around individual embryos and the entire stack of green channel was converted to a TIFF file. In Ilastik, a single bright embryo slice was chosen in each replicate experiment, and the foreground and background fluorescence were delineated. All the other TIFF files were then imported and calibrated against this standard. For each embryo, all tissue slices were summed from 16 to 18 5 µm Z-stack slices to give an individual embryo value. The average fluorescence of the negative control embryos was subtracted, embryos that had lower fluorescence than the negative were set to 0. For each antibody, there were at least two (OCT4 and CDX2), three (YAP, Na^+^/K^+^ ATPase alpha1 subunit or E-cadherin) or four (ZO-1) different embryo collections subjected to separate staining and quantification protocols. There was a minimum of 22 to a maximum of 68 embryos evaluated per treatment.

### Statistical analysis

GraphPad PRISM (https://www.graphpad.com/scientific-software/prism/) was employed to perform statistical analyses. For cell fate protein localization studies, each embryo examined represented a single *n* value within a treatment. However, for assessments of developmental stage, a biological replicate consisted of a single pool of embryos within a treatment group. Blastocyst development was analyzed using a one-way ANOVA test, and means were compared to one another by an *ad hoc* Tukey’s multiple comparisons test. Data analysis for DAPI-, OCT4-, CDX2- and YAP-positive nuclei included applying Kruskal–Wallis non-parametric ANOVA (as values were not normally distributed) with Dunn’s multiple comparisons tests. Data analysis of Na+/K+ ATPase, ZO-1 and E-cadherin fluorescence intensity was performed with non-parametric Kruskal–Wallis ANOVA with Dunn’s multiple comparisons tests. For all tests, *P* values of less than or equal to 0.05 were considered significantly different from one another.

## Results

### PA alone reduces blastocyst formation and total cell number while development is restored in combination with OA development

**Figure 1:**
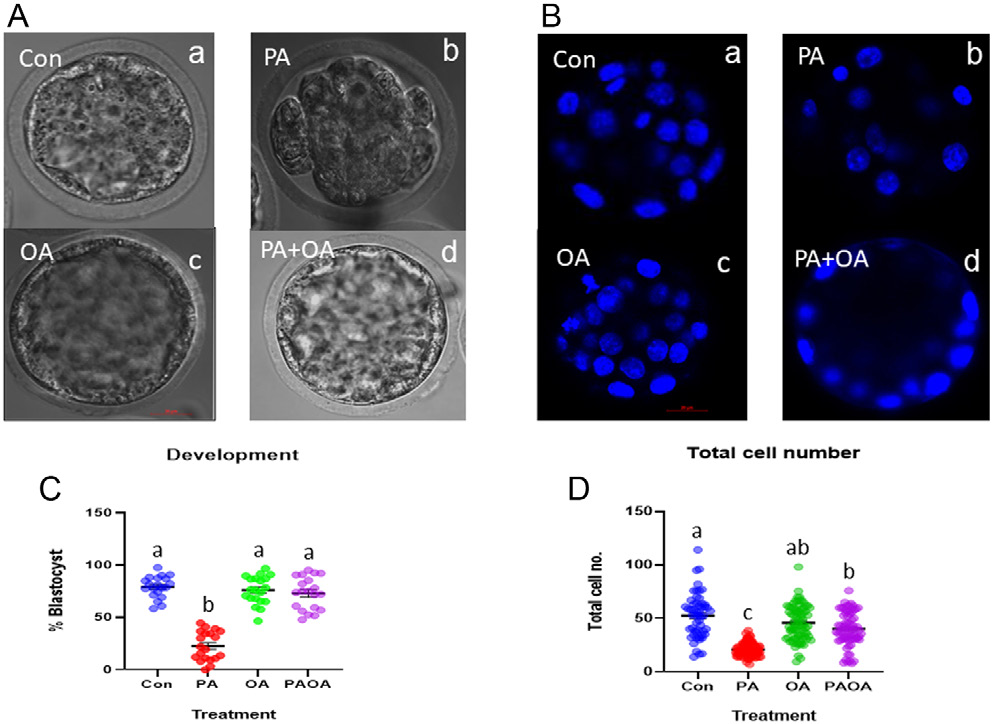
Representative phase contrast and DAPI-stained images from each treatment group. Brightfield (A) and confocal microscopy of preimplantation embryos imaged at 40× and stained with (B) DAPI (blue). (a) Control embryo, (b) PA-treated embryo, (c) OA-treated embryo and (d) PA and OA combination-treated embryo. Scale bars = 20 µm. (C) Data represent the mean percentage of blastocysts ±s.e.(*n* =20, s.e.m.). (D) The mean cell numbers ± s.e.m. observed for each treatment group. Groups that do not share the same letter are significantly different, *P*  < 0.05. (A, B, D) Control embryos (*n* = 5 experimental replicates, 9–13 embryos per replicate), PA-treated embryos (*n* = 5, 11–36 embryos per replicate), OA-treated embryos (*n* = 5, 13–21 embryos per replicate) and PA and OA combination-treated embryos (*n* = 5, 7–19 embryos per replicate).

Preimplantation embryos from each treatment group were assessed using conventional light microscopy. Blastocysts were identified as having fluid-filled cavities (Fig. 1A) and were quantified as a percentage of all preimplantation embryos observed in each culture treatment group (Fig. 1C). A statistically significant decrease (*P*  < 0.001) in the number of blastocysts was observed in the PA-treated group compared to all other groups (Fig. 1C), which is consistent with our previous study (Yousif *et al.* 2020). There were no significant differences in blastocyst developmental frequency between the control, OA alone and PA and OA combination (PA/OA) treatment groups (Fig. 1C).

**Figure 2:**
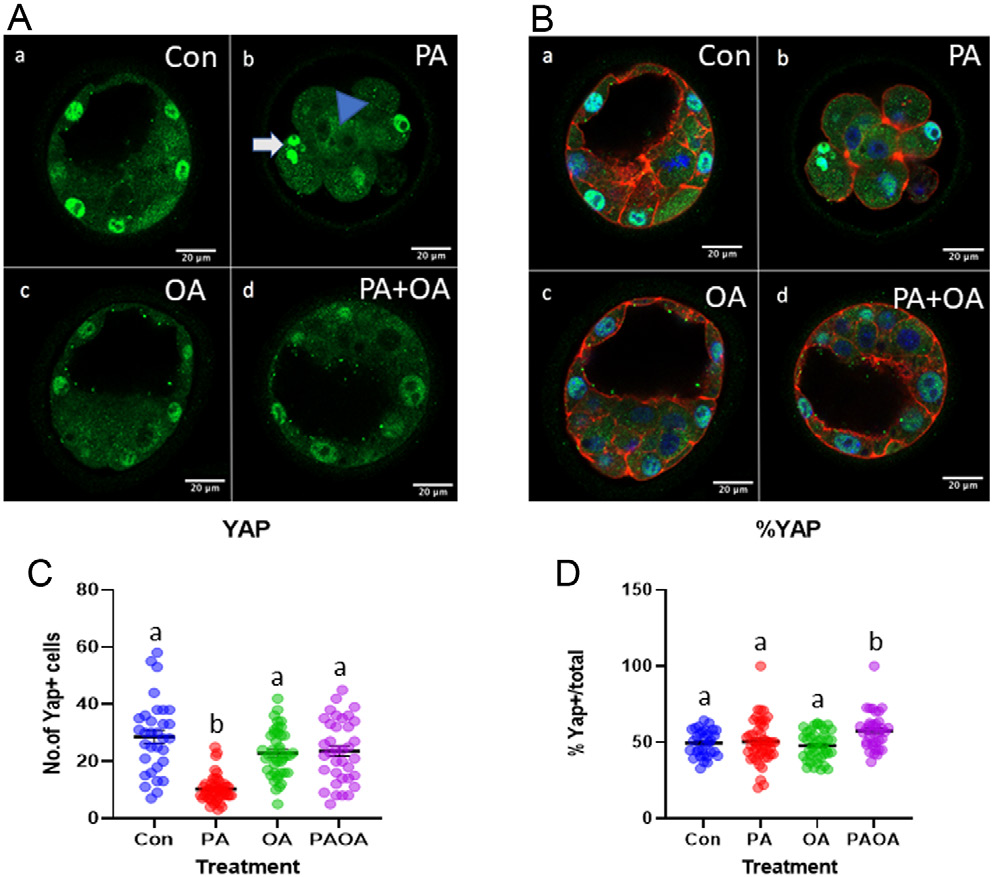
Representative YAP immunofluorescence images from each treatment group. OA attenuates the PA-induced decrease in number of cells expressing nuclear YAP. Representative confocal microscopy images of preimplantation embryos imaged at 40× following processing for indirect immunofluorescence (A) YAP (green), (B) rhodamine phalloidin (F-actin, red), DAPI (blue) representative merged images. (a) Control embryo, (b) PA-treated embryo, (c) OA-treated embryo and (d) PA and OA combination-treated embryo. Scale bars = 20 µm. PA-treated embryos frequently demonstrated binucleate cells. White arrow demonstrates binucleate cell with YAP^+^ nuclei, while blue arrowhead shows a cell with binucleate YAP^-^ nuclei. Control (*n* = 3 experimental replicates, 8–16 embryos per replicate), PA treated (*n* = 3, 11–19 embryos per replicate), OA treated (*n* = 3, 13–15 embryos per replicate) and PA and OA co-treated preimplantation embryos (*n* = 3, 7–15 embryos per replicate). (C) Data shown represent the value of the individual embryos and mean number of cells with nuclear YAP ± s.e.m. in each embryo for each treatment group. (D) The value of the individual embryos and the mean proportion of nuclear YAP^+^ cells ± s.e.m. of total cells in all embryos in each treatment. Groups that do not share the same letters are significantly different, *P*  < 0.05.

We assessed the total number of DAPI-stained nuclei observed in each embryo composing each treatment group (Fig. 1B and  D). PA-treated embryos displayed a significant reduction (*P*  < 0.001) in total number of nuclei compared to all other treatment groups and control group (Fig. 1D). PA/OA had lower cell number than control (*P*  < 0.04, Fig. 1D). The majority of PA-treated embryos appeared as uncompacted cleavage stage embryos (Fig. 2Ab). The mean cell number of the PA-treated group was approximately 21 cells.

### Effects of PA and OA treatment alone and in combination of YAP^+^ nuclei

The number of cells with YAP localized to the nucleus following each treatment was counted (Fig. 2A and  C). The presence of binucleate cells in the PA-treated group was commonly observed (Fig. 2Ab and Bb). Some YAP^+^ and YAP^−^ cells were binucleate; however, cells with YAP^-^ nuclei were properly localized to the inside. PA-treated embryos displayed a significantly (*P*  < 0.0001) lower number of YAP^+^ nuclei compared to all other treatment groups (Fig. 2C). Interestingly, the proportion of YAP^+^ nuclei to total nuclei did not vary significantly (*P*  > 0.05) between the control, PA and OA treatment groups (Fig. 2D). The proportion of YAP^+^ nuclei to total cell nuclei in the PA and OA combination treatment was significantly (*P*  < 0.05) higher than that observed for the control, OA and PA alone treatments (Fig. 2D).

### PA reduced the number of OCT4^+^ nuclei, while OA in combination with PA restored OCT4^+^ number

**Figure 3:**
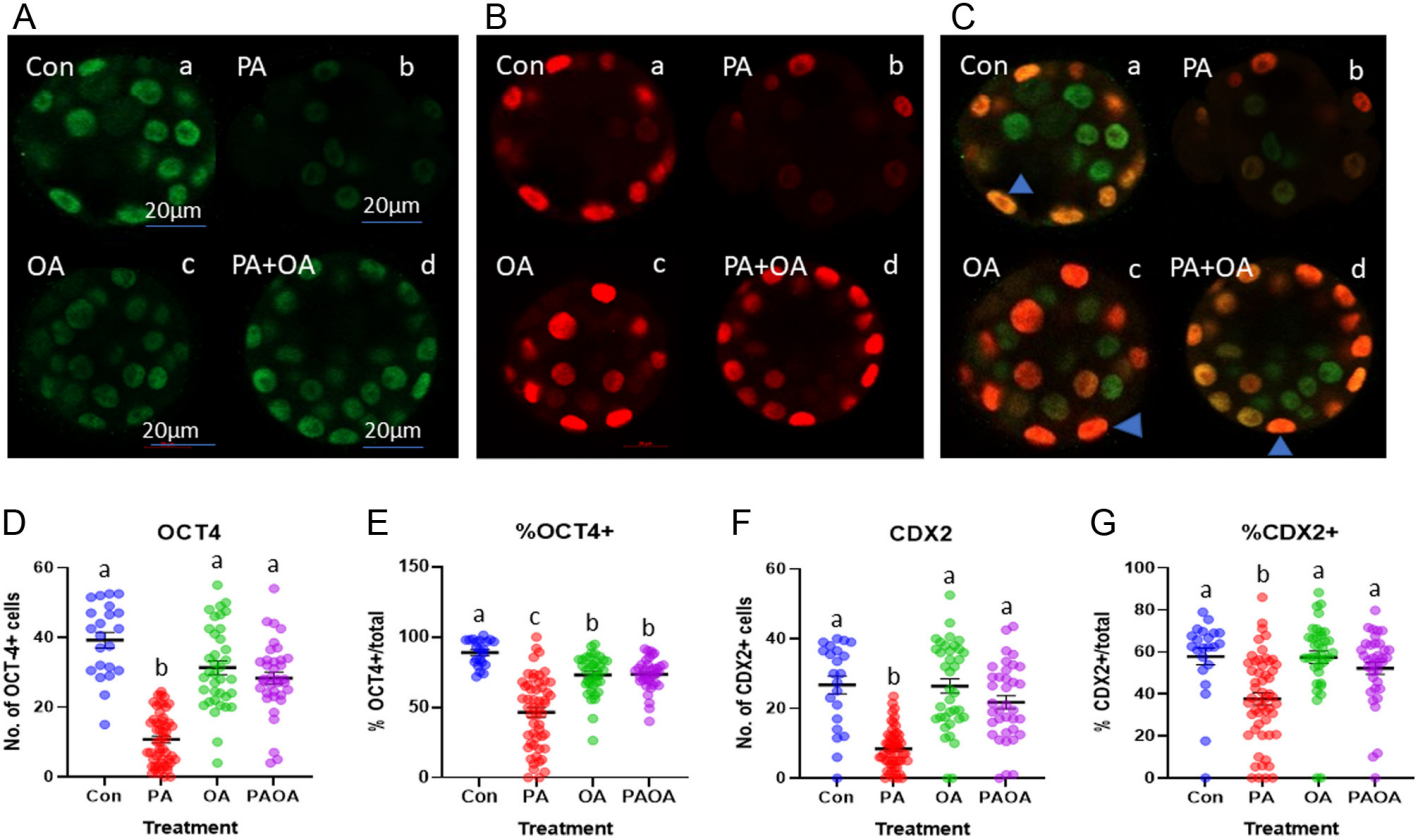
Representative OCT4 and CDX2 immunofluorescence images and quantification. Confocal microscopy images of preimplantation embryos imaged at 40× following processing for indirect immunofluorescence (A) OCT4 (green), (B) CDX2 (red), (C) merged representative OCT4 and CDX2 images. (a) Control embryo, (b) PA-treated embryo, (c) OA-treated embryo and (d) PA and OA combination-treated embryo. Scale bars = 20 µm. At early blastocyst stage, some outer nuclei still co-express OCT4 and CDX2 (yellow to orange staining), blue arrowheads. (D) Data shown represent the value of the individual embryos and the mean total OCT4^+^ nuclei ± s.e.m. for each treatment group. (E) The mean proportion of OCT4^+^ nuclei ± s.e.m. of total cells in each treatment. (F) Data shown represent the mean total CDX2^+^ nuclei ± s.e.m. for each treatment group. (G) The mean proportion of CDX2^+^ nuclei ± s.e.m. of total cells in each treatment. Groups that do not share the same letters are significantly different, *P*  < 0.05. Control embryos (*n* = 2 experimental replicates, 10–12 embryos per replicate), PA-treated embryos (*n* = 2, 18–36 embryos per replicate), OA-treated embryos (*n* = 2, 17–20 embryos per replicate) and PA and OA combination-treated embryos (*n* = 2, 17–19 embryos per replicate).

PA-treated embryos displayed a significantly lower (*P*  < 0.0001) number of OCT4^+^ nuclei compared to the vehicle-treated control group and other treatment groups (Fig. 3Ab and  D). The mean proportion of OCT4^+^ nuclei in the control, OA and PA/OA embryos was significantly greater than the PA-treated embryos (*P*  < 0.0001). The proportion of OCT4+ nuclei in OA-treated embryos (*P*  < 0.005) and PA and OA combination-treated embryos (*P*  < 0.004) was lower than control embryos (Fig. 3E). There was no significant difference in the proportion of OCT4^+^ nuclei between OA- and PA/OA-treated embryos (Fig. 3E).

### PA reduced the number of CDX2^+^ nuclei, while OA in combination with PA restored CDX2+ cell number

The number of CDX2^+^ nuclei in the PA-treated embryos was significantly lower (*P*  < 0.0001) compared to all other groups (Fig. 3Bb and  F). The mean proportion of CDX2^+^ cells in PA-treated group was significantly lower (*P*  < 0.0001) than the control and OA treatment groups and lower than the combined PA/OA group (*P*  < 0.005, Fig. 3G). Control, OA and PA/OA treatments were not significantly different from each other.

### PA reduced the amount of Na^+^/K^+^-ATPase α-1 subunit, E-cadherin and ZO-1 membrane localization

**Figure 4:**
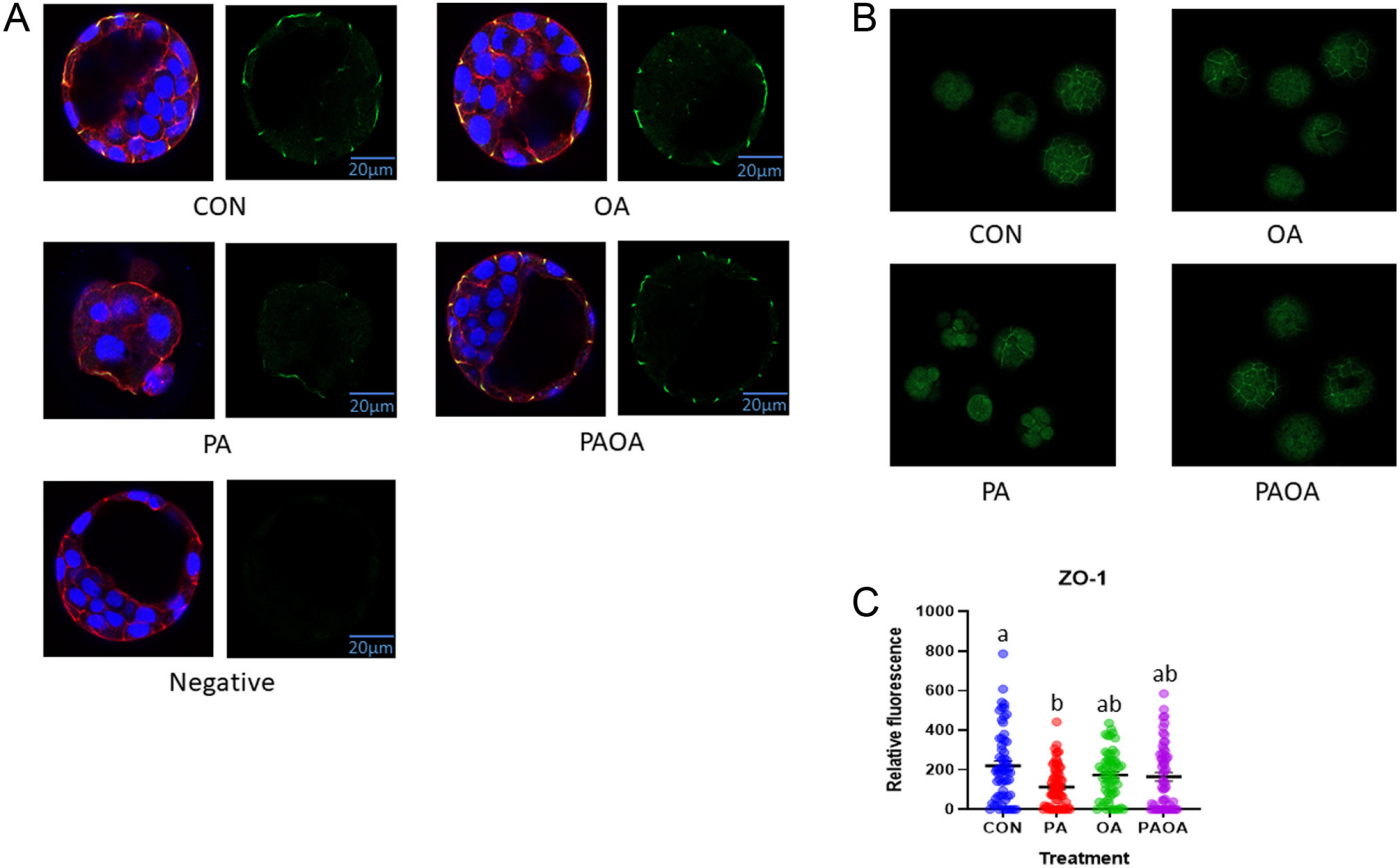
Representative Na^+^/K^+^ ATPase α-1 immunofluorescence images from each treatment group. Confocal microscopy images of preimplantation embryos imaged at 40× following processing for indirect immunofluorescence (A) Na^+^/K^+^ ATPase α-1 subunit (green), F-actin (red), DAPI (blue), merged images on left panel and Na^+^/K^+^ ATPase α-1 subunit alone on right panel. (a) Control embryo, (b) PA-treated embryo, (c) OA-treated embryo and (d) PA and OA combination-treated embryo. Scale bars = 20 µm. (B) Embryo group photo of Na^+^/K^+^ ATPase α-1 (green), 10× magnification, (C) quantification of Na^+^/K^+^ ATPase α-1 intensity in each treatment group. Data shown represent the values of the individual embryos and the mean Na^+^/K^+^ ATPase α-1 fluorescence ± s.e.m. for each treatment group. Bars without letters in common are significantly different, *P*  < 0.05. Control embryos (*n* = 3 experimental replicates, 13–16 embryos per replicate), PA-treated embryos (*n* = 3, 12–21 embryos per replicate), OA-treated embryos (*n* =3, 12–15 embryos per replicate) and PA and OA combination-treated embryos (*n* = 3, 14–15 embryos per replicate).

**Figure 5:**
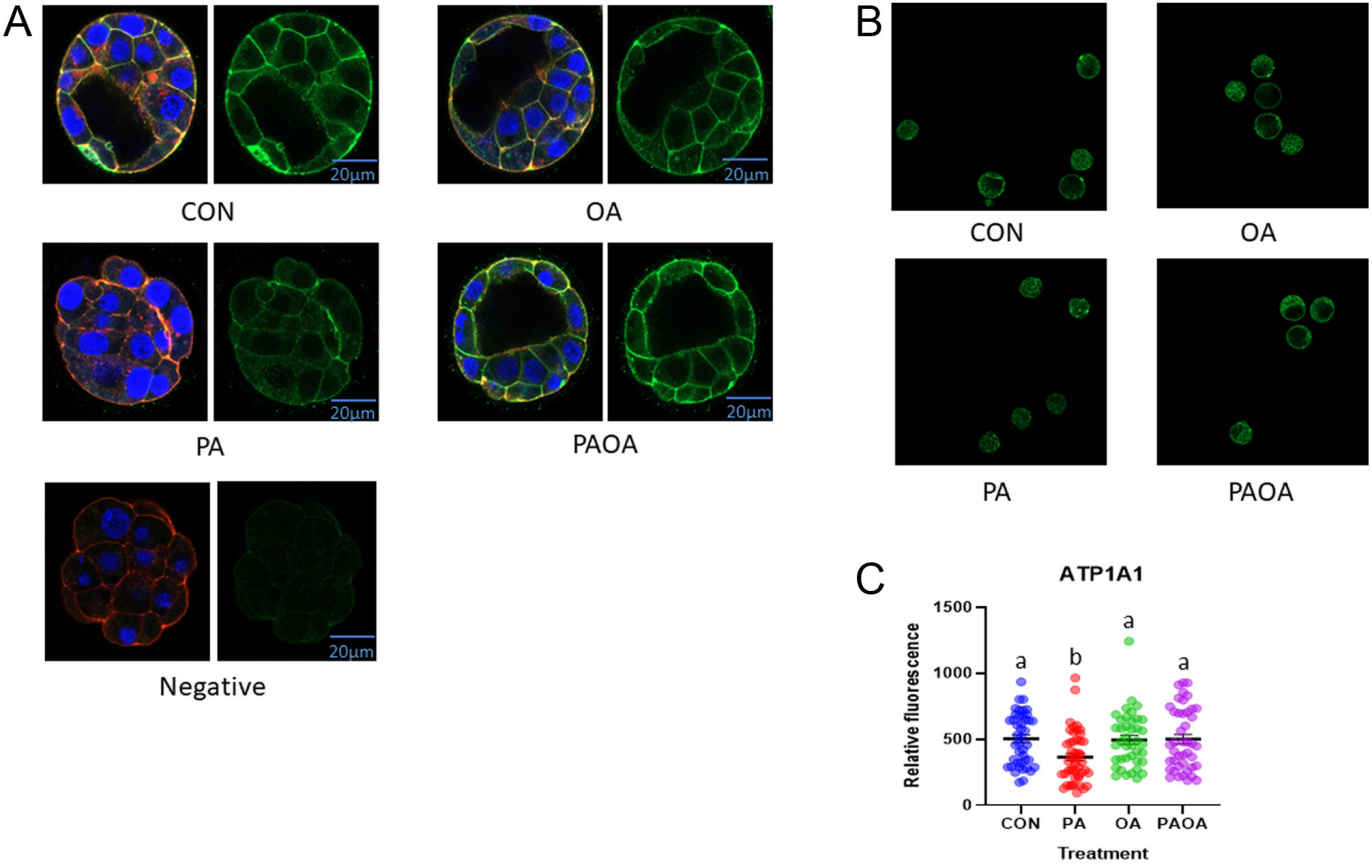
Representative E-cadherin immunofluorescence images from each treatment group. Confocal microscopy images of preimplantation embryos imaged at 40× following processing for indirect immunofluorescence (A) E-cadherin (green), F-actin (red), DAPI (blue), merged images on left panel and E-cadherin alone on right panel. (a) Control embryo, (b) PA-treated embryo, (c) OA-treated embryo and (d) PA and OA combination-treated embryo. Scale bars = 20 µm. (B) Embryo group photo of E-cadherin (green), 10× magnification (a) Control embryo, (b) PA-treated embryo, (c) OA-treated embryo and (d) PA and OA combination-treated embryo. (C) Quantification of E-cadherin intensity in each treatment group. Data shown represent the values of the individual embryos and the mean E-cadherin fluorescence ± s.e.m. for each treatment group. Bars without letters in common are significantly different, *P*  < 0.05. Control embryos (*n* = 3 experimental replicates, 15–20 embryos per replicate), PA-treated embryos (*n* = 3, 17–19 embryos per replicate), OA-treated embryos (*n* =3, 12–20 embryos per replicate) and PA and OA combination-treated embryos (*n* = 3, 14–18 embryos per replicate).

**Figure 6:**
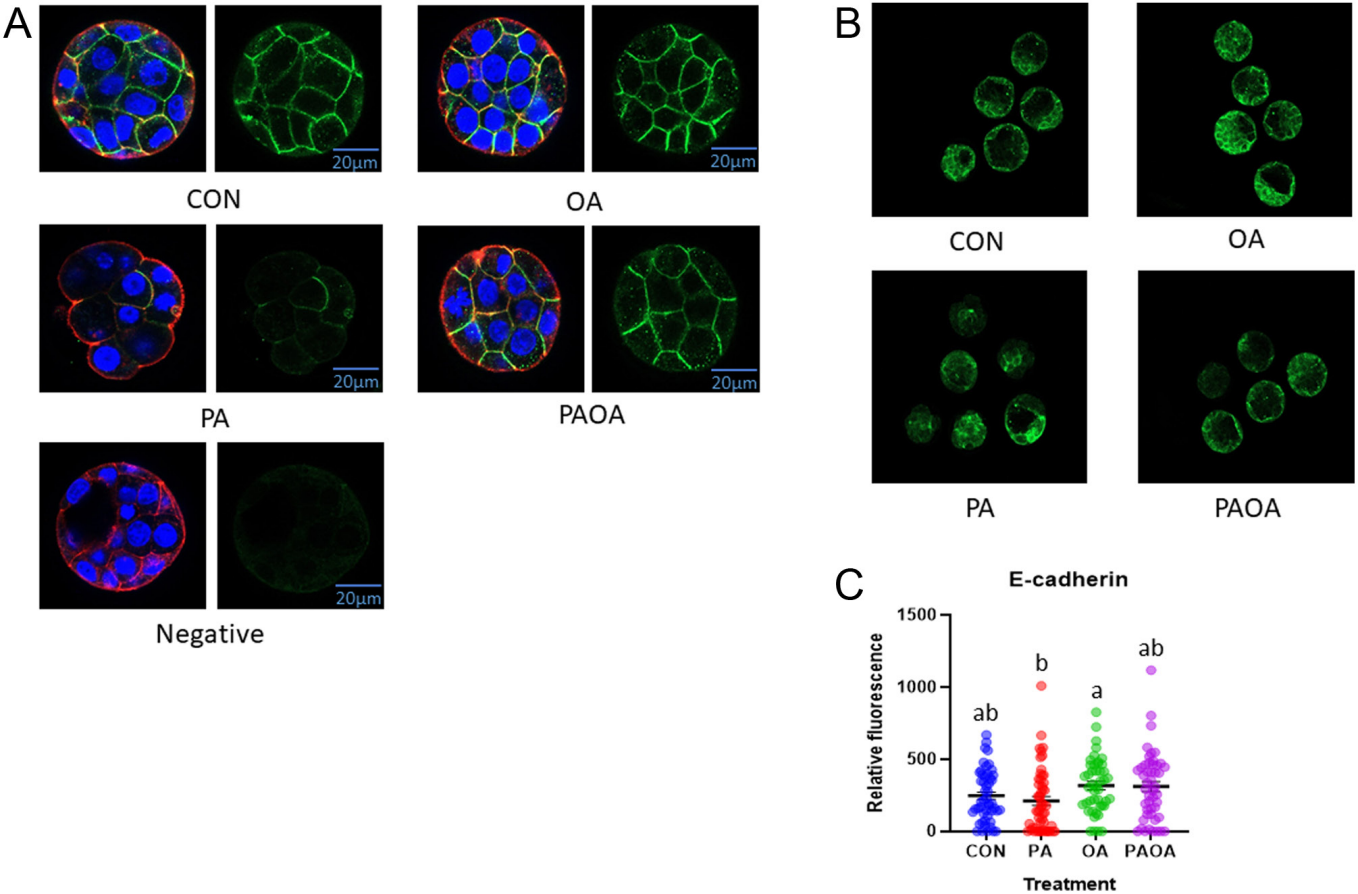
Representative ZO-1 images from each treatment group. Confocal microscopy images of preimplantation embryos imaged at 40× following processing for indirect immunofluorescence (A) ZO-1 subunit (green), F-actin (red), DAPI (blue), merged images on left panel, ZO-1 alone on right panel. (a) Control embryo, (b) PA-treated embryo, (c) OA-treated embryo and (d) PA and OA combination-treated embryo. Scale bars = 20 µm. (B) Embryo group photo of ZO-1 (green), 10× magnification. (C) Quantification of ZO-1 intensity in each treatment group. Data shown represent the values of the individual embryos and the mean ZO-1 fluorescence ± s.e.m. for each treatment group. Bars without letters in common are significantly different, *P*  < 0.05. Control embryos (*n* = 4 experimental replicates, 11–18 embryos per replicate), PA-treated embryos (*n* = 4, 10–24 embryos per replicate), OA-treated embryos (*n* = 4, 13–16 embryos per replicate) and PA and OA combination-treated embryos (*n* = 4, 13–16 embryos per replicate).

The level of Na^+^/K^+^-ATPase fluorescence intensity in PA-treated embryos was significantly lower (*P*  < 0.005) compared to CON and *P*  < 0.02 for both OA and PA/OA (Fig. 4A, B and  C). The level of E-cadherin fluorescence intensity was significantly lower (*P*  < 0.02) in PA-treated embryos compared to the OA group (Fig. 5A, B and  C). The level of ZO-1 fluorescence intensity was also significantly lower (*P*  < 0.004) in PA-treated embryos compared to the CON group (Fig. 6A, B and  C).

## Discussion

Treatment of mouse embryos with PA resulted in impaired development similar to previous studies (Jungheim *et al.* 2011*a*, Yousif *et al.* 2020). The concentrations of PA and OA used are like those of serum levels in normal pregnant women of about 100 µM (Chen *et al.* 2010), but PA can increase 30–60% in serum and follicular fluid in obese patients (Jungheim *et al.* 2011*b*, Valckx *et al.* 2014). Cleavage (O’Gorman *et al.* 2013) and pregnancy (Mirabi *et al.* 2017) in ART cycles are negatively correlated with follicular fluid PA concentrations. In the embryos treated with 100 µM PA, the cell number averaged 21 cells, many embryos arrested at the 8–16 cell stage and 22% developed to the blastocyst stage. Short-term exposure of mammalian female gametes to PA showed long-lasting effects, as development was delayed (Desmet *et al.* 2020) and fetuses and liveborn animals were smaller following embryo transfer (Jungheim *et al.* 2011*a*). As well, gene expression was altered in both embryo and extra-embryonic tissue (Desmet *et al.* 2020). ER stress pathways are activated in mammalian female gametes and granulosa cells subjected to PA treatment *in vitro* (Wu *et al.* 2012, Yousif *et al*. 2020).

We observed that PA-treated embryos developed multi-nuclear cells. Multi-nuclear blastomeres in human embryos are quite common (Hardy *et al.* 1993), but they are rare in cultured CD1 mouse embryos. In human embryos, multi-nucleation is associated with aneuploidies (Staessen & Van Steirteghem 1998). However, binucleation can resolve if it occurs at the two-cell stage (Aguilar *et al.* 2016, Paim & FitzHarris 2020) but not later (Paim & FitzHarris 2019). Paim and FitzHarris (2020) suggest that binucleate cells in later stage mouse embryos arise due to a failure of cytokinesis, as was observed earlier in arrested human embryos (Hardy *et al.* 1993). Zhang *et al.* (2010) demonstrated that PA treatment resulted in the formation of binuclear HeLa cells due to disrupted cytokinesis. Difficulties in completing cytokinesis may underlie the reduced cell numbers of the PA treatment group in the present study. Further studies are required to determine whether this is the case and when binucleation is occurring in PA-treated 2-cell embryos.

While we have an excellent understanding of the basic mechanisms and molecular players involved in cell lineage specification in mammalian embryos, there is more to learn about governing mechanisms that coordinate the timing of cell fate events and their sequence (Piliszek *et al.* 2016). Early blastomeres transition from totipotency to obtaining positional information (outside or inside cells), triggering polarization, adherens junction cell to cell adhesion, and compaction leading to epithelialization and differentiation of TE cells (Piliszek *et al.* 2016). The role of the Hippo pathway in TE differentiation is to differentially regulate YAP phosphorylation in TE and ICM development (Nishioka *et al.* 2009, Anani *et al.* 2014, Sasaki 2017). Normally following compaction at the 8-cell stage, each blastomere acquires polarity (Johnson *et al.* 1986). At the 16-cell stage, nuclear YAP is expressed in the outer cells, controlled mainly by blastomeres inheriting apical membrane components (Anani *et al.* 2014, Hirate *et al.* 2015, Sasaki 2017). In the outer cells, i.e. TE progenitors, Hippo activity is inactivated thus allowing YAP to localize to the nucleus and interact with TEA domain transcription factor (TEAD) resulting in CDX2 expression and TE cell differentiation (Nishioka *et al.* 2009, Anani *et al.* 2014, Sasaki 2017). In contrast, the Hippo pathway is active in the ICM which results in phosphorylated YAP that remains cytoplasmic (Nishioka *et al.* 2009, Anani *et al.* 2014, Sasaki 2017). By the 32-cell stage, differential activation of Hippo signaling in the two-cell lineages is cemented by the formation of stable E-cadherin-mediated adherens junctions (Nishioka *et al.* 2009, Anani *et al.* 2014, Sasaki 2017). PA-treated embryos were predominantly 8–16-cells; still, YAP immunofluorescence localization appeared to be correct for regulating cell fate in outer cells, i.e. nuclear in outer cells. The proper localization of YAP may indicate that PA treatment did not alter membrane structure or domain inheritance. Since they are chronological at a normal developmental time point for these mechanisms to reveal themselves, we suggest that the PA-treated embryos were simply activating a normal cell fate developmental program according to the polar or apolar status of the blastomeres to the best of their ability.

The number of YAP^+^ nuclei was lower in the PA group in comparison to other groups; however, the proportion of YAP+ nuclei was unaffected. PA embryos including cleavage stage embryos displayed nuclear YAP immunofluorescence. Other studies have found that PA reduced the number of endothelial cells with nuclear YAP (Yuan *et al.* 2017); however, studies with β-cell lines have shown that PA induced nuclear localization of YAP (Deng *et al.* 2016). Inhibition of YAP resulted in increased PA-induced apoptosis, suggesting that nuclear YAP may have a protective role in β-cells (Deng *et al.* 2016). Since, the proportion of YAP^+^ nuclei did not vary significantly between PA and control groups, we would propose that cell lineage specification mechanisms were activated, but for the majority of PA-treated embryos, this was not sufficient to enable cavitation and blastocyst formation.

We observed lower numbers and proportion of OCT4^+^ cells in PA-treated embryos, which is indicative of ICM. Similarly, the proportion of ICM cells was lower in embryos retrieved from high-fat diet fed mice (Minge *et al.* 2008, McPherson *et al.* 2015) and the number of ICM cells was lower in embryos from overweight women (Leary *et al.* 2015). CDX2^+^ cell numbers and proportion were also reduced in PA-treated embryos, which is indicative of reduced numbers of TE cells. Similarly, the number of TE cells is reduced in embryos from overweight women (Leary *et al.* 2015). Both decreased proliferation and increased apoptosis were observed in trophoblast stem cells cultured with PA (Jungheim *et al.* 2011*a*). The reduction in total cell number as well as both ICM and TE cells suggests that PA treatment promotes metabolic changes within the early embryo (McKeegan & Sturmey 2011). Leary *et al.* (2015) observed reductions in glucose uptake, alterations in amino acid turnover and increases triglyceride storage in embryos from overweight women.

We also observed that PA treatment reduced the fluorescent levels of Na^+^/K^+^ATPase, E-cadherin and ZO1 properly localized to blastomere cell margins. Exposure to high-fat diet reduced mRNA expression and reduced assembly of tight junctions and adherens junctions as well as Na^+^/K^+^ATPase expression and function in corneal cells (Bu *et al.* 2020). In addition, increased amounts of PA in the lipid bilayer reduced Na^+^/K^+^ATPase function (Gianfrancesco *et al.* 2019). Na^+^/K^+^ATPase activity is linked to membrane fluidity and negatively associated with levels of saturated fatty acids in erythrocytes (Rodrigo *et al.* 2014). Similarly, palmitate decreased integrity of tight and adherens junctions and increased permeability of the gut epithelium (Ghezzal *et al.* 2020). Failure to establish these junctions appropriately and produce Na/K-ATPase activity required for establishing a trans-trophectoderm ion gradient is likely the main factor underlying the reduced ability of PA-treated embryos to cavitate and form blastocysts (Barcroft *et al.* 1998, Betts *et al.* 1998, Watson & Barcroft 2001). Furthermore, blastocyst formation gene and cell fate promoting gene expression are interconnected, as CDX2-deficient embryos have deficiencies in forming and regulating trophectoderm apical tight junctions (Strumpf *et al.* 2005) and ZO-1 gene knockout decreased expression of OCT4 and CDX2 (Wang *et al.* 2008).

The total cell number in blastocysts and the proportion of ICM cells are important determinants of embryo viability following transfer (Papaionnou & Ebert 1995, Lane & Gardner 1997). However, a precise cell number is not required for blastocyst formation to occur. For example, it is possible to split embryos in half and still observe cavitation in embryos with half their normal cell number (Tarkowski 1959, Papaionnou & Ebert 1995). Since early mammalian embryos have this capacity to cavitate with significantly reduced cell number, we were compelled to determine if their reduced ability to cavitate was associated with an alteration in the expression of known blastocyst formation genes including E-cadherin, ZO-1 and Na/K-ATPase alpha 1 subunit genes. We have extensive experience with these gene products and have significantly helped with the understanding of their essential roles in driving cavitation and thus blastocyst formation (Barcroft *et al.* 1998, Betts *et al.* 1998, Watson & Barcroft 2001, Violette *et al.* 2006). The fluorescence signal of all three blastocyst formation genes was significantly reduced in 48-h PA-treated embryos. We thus conclude that overall, it is likely that PA treatment impedes proper blastocyst formation gene product expression, and while cell fate gene products are properly localized in PA-treated embryos, their reduced ability to cavitate could be due to a disruption in normal E-cadherin, ZO-1 and Na/K-ATPase alpha subunit expression.

In conclusion, our study has reinforced the harmful effects of PA exposure on mouse embryo preimplantation development. Our observations are applicable of understanding the consequences that obesity may impose on human preimplantation embryo development as FFA within follicular fluid is positively correlated with BMI (Valckx *et al.* 2014) and negatively correlated with oocyte quality (Jungheim *et al.* 2011*b*). PA and other saturated fatty acids in the Western diet may hinder the growth of the embryo and lead to lower conception rates. In this study, treatment of two-cell preimplantation mouse embryos with 100 µM PA significantly reduced total embryo cell number and the numbers of YAP^+^, OCT4^+^, and CDX2^+^expressing nuclei as well as decreasing cell adhesion, tight junction and Na^+^/K^+^ATPase proteins in the blastomere membranes. In contrast, OA co-treatment alleviates these effects of PA treatment. There are many other FFAs that should be investigated for their influence on preimplantation development, and as progress continues in this manner, eventual understanding of the full possible effects of FFA exposure to early development will unfold. It will be important to conduct further experiments that are directed at dissecting stage-specific and treatment time course variation effects of PA and OA treatment on preimplantation development. Our findings offer potential therapeutic interventions as supplementation of embryo culture medium with OA may improve development in obese patients with elevated PA serum levels.

## Declaration of interest

The authors declare that there is no conflict of interest that could be perceived as prejudicing the impartiality of the research reported.

## Funding

This work was supported by a grant from the Canadian Institutes of Health Researchhttp://dx.doi.org/10.13039/501100000024 (CIHR) to D H B, B A R and A J W.

## Author contribution statement

R C, A M, Z C L L, S A, S C, Z M and M D C performed acquisition of data, analysis and interpretation; M D C, A J W assisted with experimental conception and design; M D C, R C, A J W and D H B drafted the manuscript; M D C, A J W, D H B and B A R final approval of article.
